# Efficacy and safety of CalliSpheres drug-eluting bead bronchial arterial infusion chemoembolization vs bland embolization in advanced lung cancer with hemoptysis: A multicenter retrospective study

**DOI:** 10.17305/bb.2024.10808

**Published:** 2024-11-13

**Authors:** Song Liu, Maoli Yin, Song Liu, Huichao Xu, Guangji Yu, Xianchuang Liu, Guimin Chen, Weiwei Zhang

**Affiliations:** 1 Department of Interventional Radiology, Affiliated Hospital of Jining Medical College, Jining, China; 2 Tumor Center, Linyi Tumor Hospital, Linyi, China; 3 Endoscopic Minimally Invasive Diagnosis and Treatment Center, Linyi People’s Hospital Intersection of Wohushan Road and Wuhan Road in Beicheng New Area, Linyi, China

**Keywords:** Malignant lung tumors, massive hemoptysis, bronchial arterial infusion chemoembolization, drug-eluting bead embolization microspheres

## Abstract

Massive hemoptysis is a life-threatening complication in patients with advanced primary lung cancer, and effective, safe treatments are crucial. This study aimed to investigate the efficacy and safety of CalliSpheres drug-eluting bead bronchial arterial infusion chemoembolization (DEB-BACE) for managing this condition. A retrospective analysis included 144 patients with advanced primary lung cancer and massive hemoptysis treated at multiple hospitals from January 2019 to January 2023. Patients undergoing bronchial artery embolization were divided into two groups: the observation group (*n* ═ 76) received CalliSpheres DEB-BACE with epirubicin, and the control group (*n* ═ 68) received 8spheres blank embolization. Both groups achieved successful hemostasis, with no statistically significant difference in success rates (observation group: 88.16%, control group: 86.76%). However, the observation group had a significantly longer median duration without hemoptysis (96 days vs 50 days). Two months post-therapy, the observation group showed higher objective response rates (82.89% vs 38.24%) and disease control rates (92.11% vs 66.18%) compared to the control group. Adverse reactions were manageable and similar between groups, with no serious complications observed. By January 31, 2024, the observation group had significantly longer median overall survival (11 months vs 7 months). The DEB-BACE treatment demonstrates safety and efficacy in managing massive hemoptysis in patients with advanced lung cancer. However, the superiority of this approach over bland embolization remains to be established through well-designed prospective studies. Future research is anticipated to provide a definitive comparison and further validate the role of DEB-BACE in clinical practice.

## Introduction

Massive hemoptysis is a prevalent emergency in lung cancer and ranks among the leading causes of mortality in patients with advanced disease. This condition is critical and dangerous, frequently resulting in death due to shock and asphyxia [1]. Bronchial artery embolization (BAE) is recognized as one of the most effective treatments for lung cancer associated with hemoptysis and has been widely adopted in clinical practice [2]. However, traditional embolization materials, such as gelatin sponge particles and polyvinyl alcohol (PVA) microspheres, often result in a low tumor necrosis rate and a high incidence of re-bleeding. These limitations adversely affect the control of hemoptysis, tumor remission rates, and long-term survival of patients [3].

To overcome these challenges, we have explored a novel embolization material: drug-loaded embolization microspheres. When utilized in bronchial arterial infusion chemoembolization (BACE), this approach is termed drug-eluting beads BACE (DEB-BACE). This method offers dual functionalities, encompassing both vascular embolization and a sustained, controlled release of chemotherapy agents. The mechanism of DEB-BACE treatment operates through its dual action on tumor vasculature via drug-eluting microspheres: firstly, the microspheres physically occlude tumor blood vessels, leading to a rapid reduction in tumor volume; secondly, the chemotherapeutic agents they carry establish a localized, high-concentration drug environment that continuously inhibits tumor cell proliferation [4]. This integration of local and systemic therapy not only enhances treatment outcomes but also mitigates the side effects typically associated with systemic chemotherapy, thereby providing a new therapeutic option for patients with advanced lung cancer.

Clinical applications of BACE have yielded promising outcomes [5, 6]. As an emerging therapeutic strategy, DEB-BACE has demonstrated both efficacy and safety in numerous clinical studies involving patients with advanced lung cancer [7, 8]. This approach synergistically combines the hemostatic effects of BAE with the therapeutic benefits of drug-eluting beads, potentially revolutionizing the management of massive hemoptysis in advanced lung cancer.

The clinical significance of DEB-BACE transcends its immediate hemostatic capabilities; it signifies a strategic shift toward a more holistic treatment paradigm that addresses both acute bleeding episodes and the underlying malignancy. Given the poor prognosis associated with advanced lung cancer and massive hemoptysis, the introduction of a treatment modality that offers both immediate and long-term benefits is of paramount importance. However, there is a scarcity of reports regarding the use of DEB-BACE in treating advanced lung cancer complicated by massive hemoptysis. This study aims to investigate the safety and efficacy of DEB-BACE in the management of advanced primary lung cancer presenting with massive hemoptysis.

## Materials and methods

### Case data

**Figure 1.**
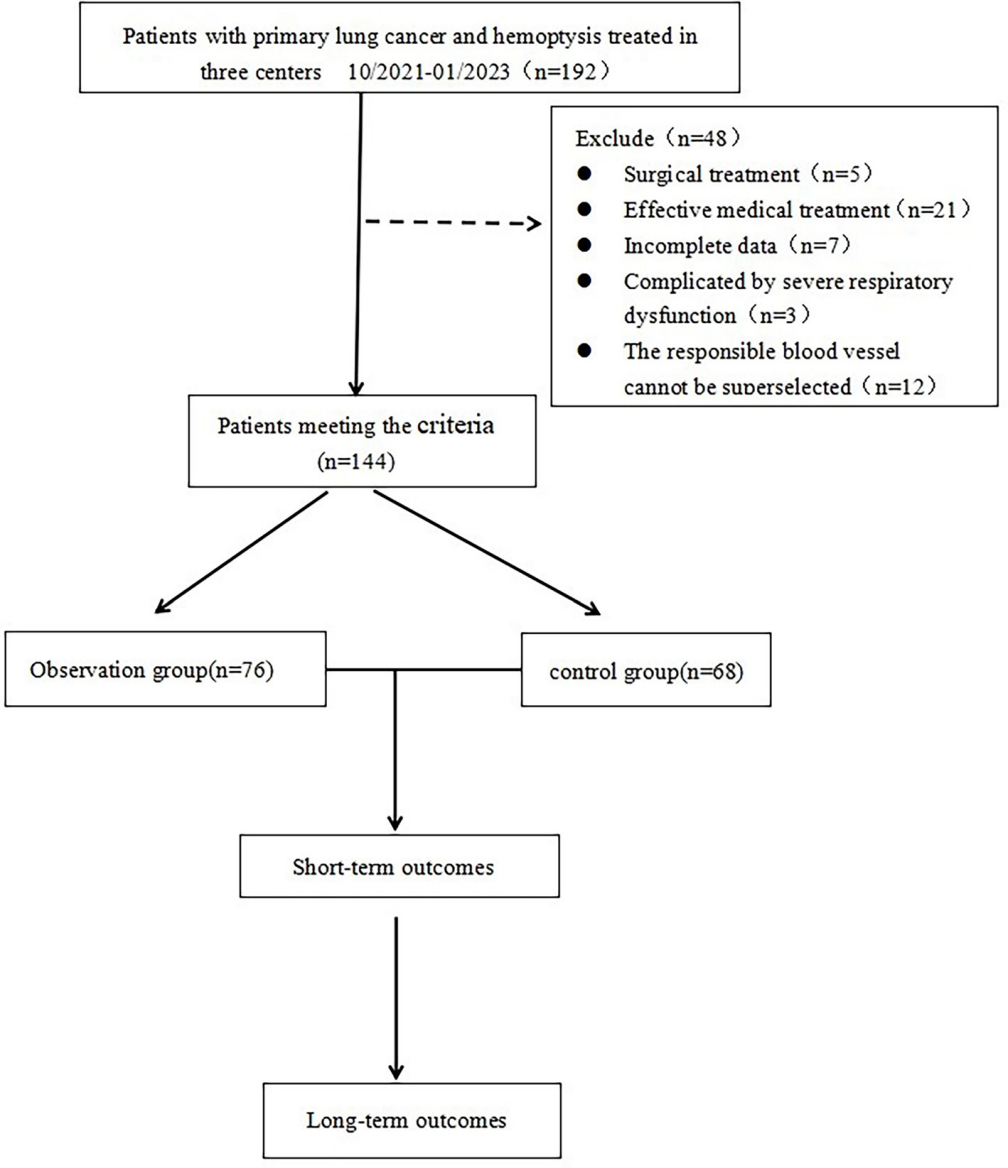
**The study flowchart for inclusion and exclusion**.

This study focused on patients with advanced primary lung cancer complicated by massive hemoptysis who were admitted to the Affiliated Hospital of Jining Medical College, Linyi Tumor Hospital, and Linyi People’s Hospital in Shandong Province from October 2021 to January 2023. The inclusion criteria were as follows: (1) patients aged 18–80 years, regardless of gender; (2) diagnosis of stage IIIc/IV primary lung cancer with massive hemoptysis (defined as hemoptysis > 500 mL in 24 h or > 100 mL in a single episode) based on medical history, clinical manifestations, and auxiliary examinations; (3) no systemic chemotherapy or radiotherapy prior to interventional therapy; (4) computed tomography angiography (CTA) or angiography indicating involvement of bronchial or other systemic arteries supplying the tumor. Some emergency cases bypassed CTA and received immediate angiography and embolization; (5) Karnofsky performance status (KPS) score ≥ 60; (6) an expected survival time of over three months. Exclusion criteria included: (1) severe cardiopulmonary or coagulation function abnormalities; (2) requirement for endotracheal intubation and artificial ventilation during the procedure; (3) active infections, iodine allergies, or contraindications to angiography; (4) tumor feeding arteries sharing a common trunk with the spinal artery, which could not be selectively embolized. In cases of lung cancer with hemoptysis, the responsible vessels primarily originate from the bronchial arteries, accounting for up to 90% of instances, which differs from hemoptysis caused by conditions such as lung abscesses or tuberculosis. These situations were also excluded from this study. The inclusion and exclusion criteria are illustrated in Figure 1. A total of 144 patients were enrolled. The treatment team, after thorough preoperative discussions considering each patient’s specific clinical characteristics, determined the treatment plan (DEB-BACE or BAE) based on the patient’s preferences and financial considerations. Patients were subsequently divided into an observation group (76 cases) and a control group (68 cases) based on the type of interventional embolization materials used. There were no significant differences in age, gender, tumor location, or tumor size between the two groups (all *P* > 0.05). For a detailed breakdown of patient demographics, tumor characteristics, and group comparisons, refer to Table 1.

**Table 1:** Comparison of general information between the two groups of patients [*x*±s/*n*(%)]

**Clinical characteristics**	**Observation group (*n* ═ 76)**	**Control group (*n* ═ 68)**	***t*/*χ*^2^ value**	***P* value**
Age	55.37±10.59	56.65±11.91	0.557	0.289
Gender			0.354	0.552
Male	57	48		
Female	19	20		
Pathological type			1.932	0.681
Squamous cell carcinoma	60	48		
Adenocarcinoma	13	14		
Small cell lung cancer	3	6	0.455	0.455
Tumor diameter (cm)	4.78±1.27	4.65±1.53		
Extrapulmonary metastasis			0.448	0.503
Yes	51	42		
No	25	26		
Tumor staging			0.005	0.945
Stage IIIc	16	14		
Stage IV	60	54		
Location of the tumor			0.612	0.433
Central type	70	60		
Peripheral type	6	8		
Previous antineoplastic therapy			0.350	0.553
Yes	67	62		
No	9	6		

### Treatment

#### Control group (BAE)

Under local anesthesia, a modified Seldinger technique was employed to puncture the right femoral artery. A 5F pigtail catheter was introduced into the thoracic aorta via a 5F vascular sheath to capture images of the thoracic aorta, followed by the use of 5F Cobra, MIK, and TIG catheters for bronchial arteriography. Angiography of intercostal arteries, internal thoracic arteries, branches of the thyrocervical trunk, phrenic arteries, and left gastric arteries was performed as necessary to identify the responsible vessels. A microcatheter (Progreat 2.7F) was then superselected to the offending artery, with confirmation that non-offending vessels, such as the spinal artery, were avoided through additional angiography. Based on angiographic results, 8 spheres (300–500 µm) were utilized for embolization, and larger microspheres (500–700 µm) were used for specific angiographic assessments. Angiography was subsequently conducted to evaluate the degree of vascular embolization, with endpoints defined as stasis or near stasis in the embolizing vessel or significant loss of tumor staining.

#### Observation group

The procedures for puncture catheterization, arteriography, and superselection of offending vessels using the microcatheter (Progreat 2.7F) mirrored those of the control group. Preparation of CalliSpheres Drug-Eluting Microspheres: CalliSpheres drug-eluting microspheres (1 g/vial, Suzhou Hengrui Kalisen Biopharma Co., Ltd., National Medical Products Administration of China approval number 20153771072) were aspirated with saline using a 20 mL syringe. The syringe was then positioned vertically for 2–3 m to allow the microspheres to settle, after which the supernatant was expelled as completely as possible. Epirubicin hydrochloride (40–60 mg) was dissolved in 5 mL of sterile water for injection. A three-way stopcock connected the syringe containing the microspheres (20 mL) to the one containing the epirubicin solution (5 mL), allowing for slow injection of the epirubicin solution into the syringe with the microspheres. The syringe, now containing both the microspheres and the chemotherapeutic agent, was capped with a needle hub, and the mixture was gently agitated every 5 m for a total of 30 m to facilitate drug absorption. The epirubicin-loaded microspheres were then mixed with a non-ionic contrast agent, iohexol, at a 1:1 ratio and allowed to stand for 5 m before use. After superselection of the offending artery, the drug-loaded microspheres were injected intermittently and slowly into the artery. Angiography was then performed to assess the degree of vascular embolization, with embolization endpoints consistent with those described for the control group.

#### Postoperative management

Conventional treatments, including oxygen inhalation, blood transfusion, anti-inflammatory therapy, rehydration, and monitoring, were administered. Once the patient’s condition stabilized, they were transferred to the medical oncology department or related departments for continued anti-tumor treatment.

### Efficacy evaluation and adverse reaction observation

**Hemoptysis control:** (1) **Technical success:** Successful superselection of offending vessels and effective embolization; (2) **Clinical success:** Following BAE or BACE, hemoptysis was either completely halted or significantly reduced (partial cessation); (3) **Clinical failure:** Persistent hemoptysis without reduction post-operation, or recurrent hemoptysis during hospitalization after temporary reduction or cessation, with hospital stays ranging from 1 to 7 days; (4) **Time without hemoptysis:** Duration from surgery to recurrence of hemoptysis or patient death.

**Tumor response:** Two months after the initial treatment, chest-enhanced computed tomography (CT) was conducted, and efficacy was evaluated using the modified Response Evaluation Criteria in Solid Tumors (mRECIST). Efficacy was categorized as complete response (CR), partial response (PR), stable disease (SD), or progressive disease (PD). The objective response rate (ORR) was calculated as (CR + PR) / total cases × 100%, and the disease control rate (DCR) as (CR + PR + SD) / total cases × 100%.

**Survival:** Overall survival (OS) was defined as the duration from treatment initiation to death or the last follow-up. Adverse events were evaluated according to the National Cancer Institute (NCI) Common Terminology Criteria for Adverse Events (CTCAE) version 5.0 and graded from 0 to 4 [9]. Adverse event assessments were conducted during the hospitalization period immediately following the procedure and at the first scheduled follow-up visit. Systemic toxicities and late-onset adverse events occurring beyond this period were not routinely documented in this study.

### Follow-up

Follow-up methods included inpatient treatment, outpatient clinic visits, and telephone consultations. Each hospital visit for treatment during the treatment period was considered a follow-up visit. Post-treatment, follow-ups were scheduled every 2–3 months. The follow-up period concluded on January 31, 2024, with no cases lost to follow-up.

### Ethics approval and consent to participate

All procedures involving human participants adhered to the ethical standards set by the institutional and national research committees, as well as the 1964 Helsinki Declaration and its subsequent amendments or comparable ethical standards. Ethical approval for this study was obtained from the Medical Science Research Ethics Committee of Linyi Cancer Hospital (Approval Number: [2019]106), and the study was conducted in accordance with the approved guidelines. Informed consent was secured from all participants or their legal guardians.

### Statistical analysis

Data analysis was conducted using SPSS software (version 20.0; IBM Corp.). Categorical variables were presented as numbers and frequencies (*n*, %) and compared between groups using the χ^2^ test or Fisher’s exact test, as appropriate. Continuous variables were assessed for normal distribution via the Shapiro–Wilk test. Normally distributed data were expressed as mean ± standard deviation and compared using the independent samples *t*-test. Non-normally distributed data were presented as median with interquartile range (IQR) and compared using the Mann–Whitney *U* test. Survival curves were generated using the Kaplan–Meier method, with differences between groups assessed using the log-rank test. Univariable Cox regression was performed to identify factors associated with OS and HFS. Variables with a *P* < 0.1 in univariable analysis, along with clinically significant variables based on prior knowledge, were entered into the multivariable Cox proportional hazards regression model. The final model was adjusted for age, gender, tumor diameter, tumor location, pathological type, extrapulmonary metastasis, and tumor stage. Results from the Cox regression are presented as hazard ratios (HRs) with 95% confidence intervals (CIs). A two-sided *P* < 0.05 was considered statistically significant for all tests, except for the univariable screening criteria for the Cox model.

## Results

### Hemoptysis control status

**Figure 2.**
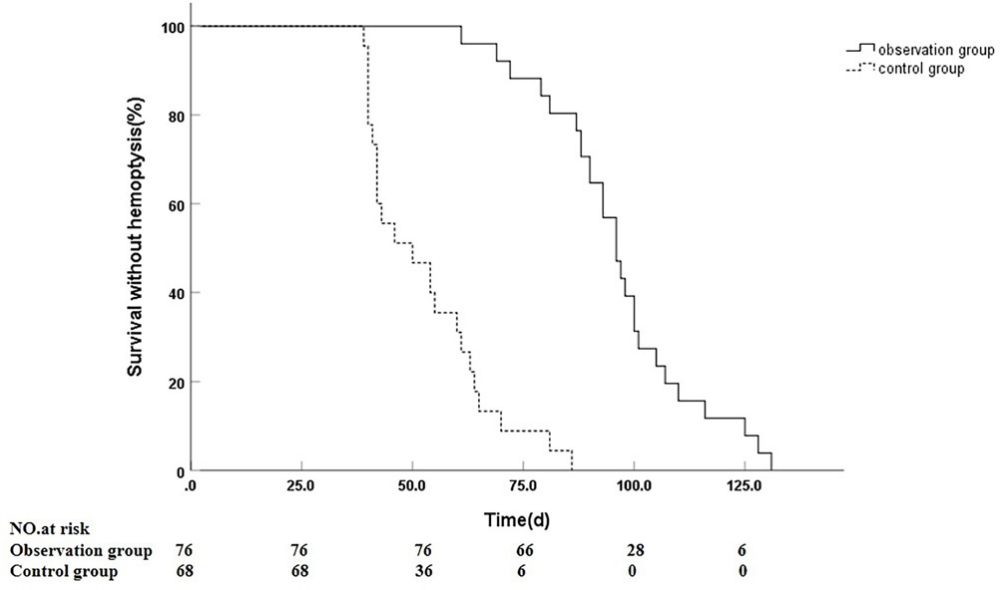
**Hemoptysis-free survival by treatment group.** Kaplan–Meier curves of hemoptysis-free survival comparing the observation group (solid line) with the control group (dashed line). The median hemoptysis-free duration was 96 days (95% CI, 92.01–99.99) in the observation group versus 50 days (95% CI, 39.69–60.31) in the control group (log-rank χ^2^ ═ 91.667, *P* < 0.001). Numbers at risk are shown below the *x*-axis. Abbreviation: CI: Confidence interval.

In total, 311 offending vessels were identified in 144 patients across both groups. The offending vessels exhibited (1) arterial tortuosity and dilation with significant tumor staining (100%, 144/144), (2) contrast agent spillover (4.17%, 6/144), and (3) arteriovenous and arterio-arterial shunting (6.25%, 9/144). The distribution of offending vessels included the left bronchial artery in 31.25% (45/144) of cases, the right bronchial artery in 56.94% (82/144), bilateral bronchial arteries in 9.72% (14/144), and other sources in 2.08% (3/144). All 144 patients underwent successful arterial embolization, yielding a technical success rate of 100% (144/144). Among these patients, 125 achieved clinical success in hemostasis, resulting in a success rate of 86.81%. This included 67 cases (88.16%) from the observation group. The clinical success rates were 86.76% for the control group and 88.16% for the observation group, with no statistically significant difference between the two groups (χ^2^ ═ 0.064, *P* ═ 0.801). Of the 19 patients who experienced failed hemostasis, four demonstrated cessation of bleeding following medical hemostatic treatment. Twelve patients underwent re-interventional procedures, with eight exhibiting recanalization of the offending vessels and subsequently undergoing re-embolization. Four patients had suspected blood supply from the ipsilateral internal mammary artery and underwent embolization, while three patients discontinued treatment and died within three days post-discharge. The median hemoptysis-free duration was significantly longer in the observation group at 96 days (95% CI: 92.01–99.99) compared to 50 days (95% CI: 39.69–60.31) in the control group (χ^2^ ═ 91.667, *P* < 0.001), as illustrated in Figure 2.

### Tumor response after interventional operation

Enhanced chest CT scans were re-evaluated two months post-intervention, and tumor response was assessed using the mRECIST. In the observation group, there were 12 cases of CR, 51 cases of PR, 7 cases of SD, and 6 cases of PD, yielding an overall response rate (ORR) of 82.89% (63/76) and a DCR of 92.11% (70/76). In contrast, the control group reported zero cases of CR, 26 cases of PR, 19 cases of SD, and 23 cases of PD, resulting in an ORR of 38.24% (26/68) and a DCR of 66.18% (45/68). The ORR and DCR in the observation group were significantly higher than those in the control group (χ^2^ ═ 30.322, *P* < 0.001; χ^2^ ═ 15.002, *P* ═ 0.001). Detailed results are presented in Table 2.

**Table 2:** Comparison of short-term clinical efficacy between the two groups [*n*(%)]

**Group**	**CR**	**PR**	**SD**	**PD**	**ORR**	**DCR**
Observation group (*n* ═ 76)	12 (15.78)	51 (67.11)	7 (9.21)	6 (7.89)	63 (82.89)	70 (92.11)
Control group (*n* ═ 68)	0 (0.0)	26 (38.24)	19 (27.94)	23 (33.82)	26 (38.24)	45 (66.18)

Abbreviations: CR: Complete response; PR: Partial response; SD: Stable disease; PD: Progressive disease; ORR: Objective response rate; DCR: Disease control rate; *n*: Number of participants.

### Survival analysis

**Figure 3.**
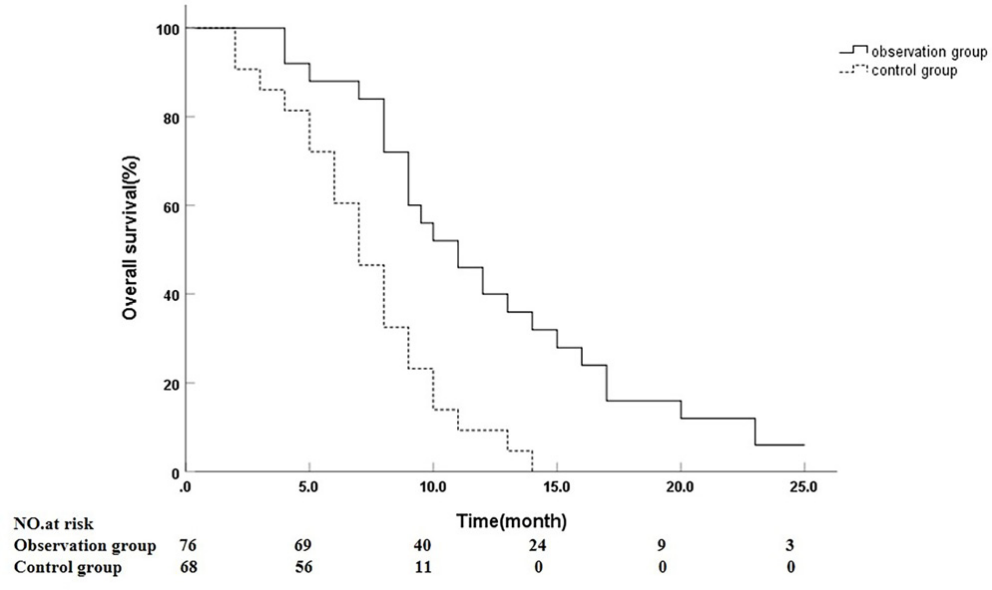
**Kaplan–Meier** **OS** **by group.** Follow-up 2–27 months. Median OS was 11 months (95% CI 8.84–13.16; *n* ═ 76) in the observation group vs 7 months (95% CI 5.93–8.07; *n* ═ 68) in controls (log-rank *P* < 0.001). Solid line = observation; dashed line = control. Numbers at risk shown below the *x*-axis. Abbreviations: OS: Overall survival; CI: Confidence interval.

As of January 31, 2024, the follow-up period for patients ranged from 2 to 27 months, with a mean duration of 9.96 ± 5.31 months. Except for three patients who were discharged and subsequently died outside the hospital due to unsuccessful interventional hemostasis, the remaining 141 patients completed the follow-up without any losses. The median OS for patients in the observation group was 11 months (95% CI: 8.84–13.16), compared to 7 months (95% CI: 5.93–8.07) for the control group, demonstrating a statistically significant difference (*P* < 0.001) (Figure 3).

### Factors associated with survival outcomes

To identify factors influencing prognostic outcomes, we conducted Cox regression analyses. Univariate analysis revealed that treatment modality (DEB-BACE vs BAE), tumor diameter, extrapulmonary metastasis, and tumor stage were significantly associated with OS, while treatment modality, pathological type, tumor diameter, tumor location, and prior therapy were significantly correlated with hemoptysis-free survival.

Multivariable analysis, after adjusting for age, gender, tumor diameter, tumor location, pathological type, extrapulmonary metastasis, and tumor stage, indicated that DEB-BACE therapy was an independent protective factor for both OS (adjusted HR = 0.45, 95% CI: 0.30–0.67, *P* < 0.001) and hemoptysis-free survival (adjusted HR = 0.48, 95% CI: 0.34–0.68, *P* < 0.001).

Moreover, the presence of extrapulmonary metastasis was identified as an independent risk factor for OS (adjusted HR = 1.55, 95% CI: 1.02–2.34, *P* ═ 0.042). Complete results of these analyses are detailed in Tables 3 and 4.

**Table 3:** Univariable and multivariable Cox regression analyses for hemoptysis-free survival

**Variable**	**Category**	**Univariable analysis**		**Multivariable analysis**	
		**HR (95% CI)**	***P* value**	**HR (95% CI)**	***P* value**
Treatment group	DEB-BACE (Ref: BAE)	0.45 (0.32–0.63)	<0.001	0.48 (0.34–0.68)	<0.001
Age	Per year increase	1.01 (0.99–1.03)	0.307	-	-
Gender	Male (Ref: Female)	1.12 (0.76–1.65)	0.567	-	-
Pathological type	NSCLC (Ref: SCLC)	0.58 (0.35–0.96)	0.033	0.72 (0.43–1.21)	0.212
Tumor diameter	Per cm increase	1.15 (1.03–1.29)	0.015	1.09 (0.97–1.22)	0.144
Extrapulmonary metastasis	Yes (Ref: No)	1.28 (0.91–1.79)	0.156	-	-
Tumor staging	Stage IV (Ref: IIIc)	1.33 (0.87–2.03)	0.185	-	-
Location of the tumor	Central (Ref: Peripheral)	1.85 (1.05–3.28)	0.034	1.42 (0.79–2.55)	0.241
Previous therapy	Yes (Ref: No)	1.65 (1.02–2.68)	0.042	1.38 (0.67–2.08)	0.203

Abbreviations: HR: Hazard ratio; CI: Confidence interval; DEB-BACE: Drug-eluting bead bronchial artery chemoembolization; BAE: Bronchial artery embolization; NSCLC: Non-small cell lung cancer; SCLC: Small cell lung cancer.

**Table 4:** Univariable and multivariable Cox regression analyses for overall survival

**Variable**	**Category**	**Univariable analysis**		**Multivariable analysis**	
		**HR (95% CI)**	***P* value**	**HR (95% CI)**	***P* value**
Treatment group	DEB-BACE (Ref: BAE)	0.52 (0.36–0.75)	<0.001	0.45 (0.30–0.67)	<0.001
Age	Per year increase	1.02 (1.00–1.04)	0.178	-	-
Gender	Male (Ref: Female)	1.15 (0.75–1.78)	0.523	-	-
Pathological type	NSCLC (Ref: SCLC)	0.88 (0.55–1.41)	0.598	-	-
Tumor diameter	Per cm increase	1.18 (1.03–1.35)	0.024	1.15 (1.00–1.32)	0.064
Extrapulmonary metastasis	Yes (Ref: No)	1.62 (1.09–2.41)	0.019	1.55 (1.02–2.34)	0.042
Tumor staging	Stage IV (Ref: IIIc)	1.78 (1.05–3.01)	0.033	1.65 (0.96–2.84)	0.065
Location of the tumor	Central (Ref: Peripheral)	1.32 (0.72–2.42)	0.374	-	-
Previous therapy	Yes (Ref: No)	1.25 (0.73–2.16)	0.415	-	-

Abbreviations: HR: Hazard ratio; CI: Confidence interval; DEB-BACE: Drug-eluting bead bronchial artery chemoembolization; BAE: Bronchial artery embolization; NSCLC: Non-small cell lung cancer; SCLC: Small cell lung cancer.

### Adverse reactions

The primary intervention-related adverse reactions in both groups included chest pain (39/144), followed by fever (33/144). Other adverse reactions, such as cough, nausea and vomiting, and fatigue, were all graded ≤ II, with no occurrences of Grade III–IV adverse reactions. All symptoms were significantly alleviated within three to seven days following symptomatic treatment. No severe complications, such as spinal cord injury or ectopic cerebral embolism, were reported. The incidence rate of adverse reactions did not differ significantly between the two groups (all *P* > 0.05). For further details, refer to Table 5.

**Table 5:** Incidence of treatment-related adverse reactions in the two groups [*n*(%)]

**Adverse reaction**	**Observation group (*n* ═ 76)**				**Control group (*n* ═ 68)**			***χ*^2^ value**	***P* value**
	**Grade I∼II (*n*)**	**Grade III∼IV (*n*)**	**Incidence (%)**		**Grade I∼II (*n*)**	**Grade III∼IV (*n*)**	**Incidence (%)**		
Fever	19	0	25.00		14	0	20.59	0.395	0.529
Chest pain	22	0	28.95		17	0	25.00	0.283	0.595
Cough	16	0	21.05		11	0	16.18	0.560	0.454
Nausea/vomiting	9	0	11.84		6	0	8.82	0.087	0.768
Fatigue	6	0	7.89		3	0	4.41	0.743	0.389

## Discussion

The literature indicates that tumor lesions in lung cancer patients can damage pulmonary vessels, leading to ruptures in vessel walls and resulting in hemoptysis [10, 11]. In cases of lung cancer accompanied by hemoptysis, traditional treatment typically involves conservative measures such as pharmacological hemostasis; however, bleeding often recurs shortly after intervention. Surgical options are frequently unfeasible due to the tumor’s advanced stage, the patient’s poor physical condition, significant trauma, and elevated risk. BAE is a minimally invasive interventional technique that involves injecting embolic agents into the implicated artery to occlude the bleeding vessel and achieve hemostasis [12]. Conventional embolic materials, such as gelatin sponges, coil springs, and PVA particles, primarily aim to stop bleeding with minimal impact on the tumor. Nevertheless, the persistence and growth of the tumor remain the primary causes of recurrent hemoptysis [13]. Therefore, to improve hemostasis and simultaneously control tumor growth, leading to necrosis and shrinkage, it is essential to prolong patient survival and enhance quality of life [14].

Building upon the principles of BAE, BACE incorporates chemotherapeutic agents to maintain elevated drug concentrations within tumor tissues for extended periods, thereby enhancing the cytotoxic effects against tumors. Recently, BACE has emerged as a promising therapeutic modality for advanced lung cancer, especially in patients with refractory cases presenting symptoms of compression, hemorrhage, or disease progression after multiple treatment lines [15]. BACE has demonstrated notable efficacy, significantly improving both patient quality of life and survival rates. DEB-BACE represents an innovative interventional treatment modality for lung cancer. The drug-eluting beads not only permanently occlude the tumor vessels but also gradually release chemotherapeutic agents to destroy tumor cells, aiming to control tumor growth [16] and improve clinical symptoms [17]. CalliSpheres beads, an indigenous drug-eluting bead developed in China, have been utilized in lung cancer treatment. A patient with locally advanced squamous cell lung cancer achieved a pathological CR (pCR) following DEB-BACE, followed by curative surgical resection, suggesting that DEB-BACE could serve as a neoadjuvant treatment option for locally advanced non-small cell lung cancer (NSCLC) [18]. In a retrospective study by Liu et al., BACE outperformed systemic chemotherapy in terms of tumor treatment effects and quality of life for patients with advanced NSCLC [19]. Fu and colleagues studied 36 lung cancer patients experiencing hemoptysis, comparing one group receiving conventional bronchial arterial chemoembolization (cBACE) with another receiving DEB-BACE. They found that DEB-BACE achieved higher tumor response rates and longer hemoptysis-free survival compared to cBACE. It is important to note that lipiodol, a liquid embolic agent, is not recommended for BAE due to its small particle size, which can damage the bronchial wall or esophagus and lead to severe complications. In our study, we selected blank microspheres as the control embolic material, which has demonstrated higher safety in BACE management. Our efficacy assessment indicated that results in the control group patients were superior to those reported in the comparison group, suggesting that microspheres may be more suitable for BAE. Notably, the results from the observation group were largely consistent with findings from two other studies [14]. Li’s research indicates that after DEB-BACE treatment for NSCLC, rapid tumor necrosis and effective reduction in tumor burden occur, with the addition of immune checkpoint inhibitors further enhancing tumor control efficacy [20]. Our center was among the first in China to implement BACE treatments using CalliSpheres beads. In one of our prospective studies involving 21 patients with intractable, recurrent NSCLC, the ORR was 88.37%, the DCR was 95.35%, and the median survival time was 11.5 months [21]. However, reports on the use of DEB-BACE for treating lung cancer with massive hemoptysis are limited.

In this study, patients with advanced primary lung cancer and massive hemoptysis were treated with either DEB-BACE or BAE alone. The findings revealed that both groups achieved similar short-term success rates in controlling hemoptysis. However, the observation group experienced a significantly longer duration without hemoptysis compared to the control group, along with markedly higher ORR and DCR. Both DEB-BACE and BAE aim to embolize the responsible blood vessels, reflecting similar technical approaches. However, the distinct tumor control outcomes underscore the effective necrosis achieved by DEB-BACE. Short-term postoperative CT scans of some patients in the observation group revealed significant low-density necrosis and cavity-like changes in the lung tumors, indicating that DEB-BACE effectively embolizes the tumor-supplying arteries. Furthermore, the favorable drug-carrying properties of microspheres contribute to the efficacy of tumor necrosis. The interaction between microspheres and chemotherapy drugs enhances the therapeutic effect, achieving the dual goals of controlling bleeding and tumor growth. A prospective cohort study comparing DEB-BACE with cBACE in stage II–IV lung cancer patients demonstrated that DEB-BACE yielded better progression-free survival and OS rates [22]. In our current study, the median OS for patients in the observation group was 11 months, which is lower than reported in some studies [23, 24]. This discrepancy is primarily attributed to differences in baseline patient characteristics, notably a higher proportion of late-stage cases. Additionally, massive hemoptysis can exacerbate the overall condition, leading to a poorer prognosis, although the OS remains better than that of the control group. DEB-BACE facilitates the delivery of high concentrations of chemotherapeutic agents directly to tumor tissues, thereby slowing tumor progression and reducing the likelihood of recurrent bleeding by diminishing tumor angiogenesis. Furthermore, DEB-BACE shows a favorable effect in slowing tumor progression, potentially contributing to prolonged survival in lung cancer patients. No severe complications, such as ectopic embolism, were reported in either group in this study. The use of microspheres with diameters of 300–500 µm for embolization in lung cancer patients with massive hemoptysis was deemed safe, consistent with diameters reported in current literature [25, 26]. The main postoperative adverse reactions were chest pain and fever, classified as Grade I–II, with no Grade III–IV reactions observed. These reactions were attributed to post-embolization tumor swelling and necrosis and were alleviated shortly after medical intervention.

This study further validated the clinical significance of DEB-BACE therapy through multivariable Cox regression analysis. The results indicated that after adjusting for various potential confounding factors—including age, gender, tumor characteristics, and metastatic status—DEB-BACE remained an independent predictor of improved OS and hemoptysis-free survival. This finding underscores the efficacy and independent clinical relevance of DEB-BACE as a locoregional intervention for patients with advanced lung cancer complicated by hemoptysis. Notably, while univariable analysis suggested associations between factors such as tumor location or pathological type and hemoptysis control, these correlations were not significant in the multivariable model. This suggests that the therapeutic benefit of DEB-BACE may surpass and potentially override the prognostic influence of these conventional factors. Conversely, extrapulmonary metastasis was confirmed as an independent risk factor for OS, aligning with previous studies and emphasizing the need for combined systemic therapy and locoregional intervention in patients with metastatic disease.

While this study adjusted for potential confounding factors through multivariable Cox regression analysis and further validated the independent prognostic value of DEB-BACE therapy, it is essential to acknowledge the inherent limitations of its retrospective design. The non-randomized nature of this study may still be subject to unmeasured confounders and selection bias. Although stratified analysis was employed during patient selection to enhance baseline comparability between groups, and multivariable adjustments were incorporated into the statistical analyses, the possibility of residual confounding cannot be entirely ruled out. Furthermore, adverse event monitoring was primarily conducted during the immediate post-procedural period following the initial BACE and was largely limited to procedure-related complications. Systemic treatment-related toxicities and long-term adverse events were not systematically evaluated. While adverse events were graded according to CTCAE version 5.0 during hospitalization and at the first follow-up visit, the absence of serial toxicity assessments beyond that timeframe may have limited the identification of delayed or cumulative adverse effects, which should be addressed in future research. Additionally, as all enrolled patients were from medical institutions in Shandong Province, China, the generalizability of the findings may be somewhat restricted. Therefore, we advocate for larger-scale, multicenter prospective studies to validate the conclusions of this study and further assess the treatment effect in broader patient populations. Future research should aim to overcome the limitations of the current study through well-designed, large-sample, multicenter prospective trials, thereby providing a more comprehensive evaluation of the efficacy and safety profile of DEB-BACE across diverse patient populations and clinical settings.

## Conclusion

In summary, DEB-BACE, as a locoregional treatment approach, is safe and effective for treating advanced lung cancer with massive hemoptysis, offering a promising therapeutic option for these patients. This retrospective study underscores the need for prospective research to validate the superiority of DEB-BACE over traditional embolization methods and to explore whether specific subpopulations may benefit more significantly from this treatment.

## Supplemental data

Supplemental data are available at the following link: https://www.bjbms.org/ojs/index.php/bjbms/article/view/10808/3589.

## Data Availability

The datasets used and/or analyzed during the current study are available from the corresponding author upon reasonable request.
